# Endocrine‐Nutritional Synergy Between Sex Inversion and Microencapsulated Bioactives Enhances Growth, Intestinal Development, and Oxidative Stability in Nile Tilapia Larvae

**DOI:** 10.1111/jpn.70046

**Published:** 2026-02-10

**Authors:** Jaísa Casetta, Eliane Gasparino, Caroline Isabela da Silva, Simone Siemer, Gabriela Hernandes Cangianelli, Ricardo Souza Vasconcellos, Ricardo Pereira Ribeiro, Graciele Caroline Mari, Jaqueline Roesler, Juliana Sofientini, Diogo de Oliveira Marques, Stefania Claudino‐Silva

**Affiliations:** ^1^ State University of Maringá (PPZ ‐ UEM) Maringá Paraná Brazil; ^2^ Department of Animal Science at the State University of Maringá (DZO ‐ UEM) Maringá Paraná Brazil; ^3^ Department of Animal Science at Ingá University Center (UNINGÁ) Maringá Paraná Brazil

**Keywords:** essential oils, feed aditives, hormonal sex reversal, intestinal development, metabolic programming, organic acids

## Abstract

This study evaluated the combined effects of sex inversion and microencapsulated supplementation with organic acids and essential oils on growth performance, tissue morphology, gene expression, and oxidative status in Nile tilapia (*Oreochromis niloticus*) larvae. A 2×2 factorial design included non‐inverted (NI), sex‐reversed (I), microencapsulated‐supplemented (NI + M), and combined (I + M) groups. The trial lasted 28 days and used 2400 Nile tilapia larvae (GIFT strain), 6 days post‐hatching, stocked at 100 larvae per tank. The experimental diets included 60 mg of 17α‐methyltestosterone per kg⁻¹ of feed for sex reversal and/or 100 mg of a microencapsulated blend of organic acids and essential oils per kg⁻¹ of feed. The treatments significantly influenced zootechnical performance: weight gain was higher in sex‐reversed groups at both 14 and 28 days (*p* < 0.001). Total length differed at D14 (I > I + M ≈ NI + M > NI; *p* = 0.039) and at D28 (*p* < 0.001). Growth modeling showed distinct patterns: NI + M had the highest asymptote (A = 198.32) with slow growth, I + M an intermediate‐high asymptote (A = 123.79) with moderate growth, and NI a very low asymptote (A = 2.00) with rapid initial growth. Gene expression revealed elevated Mstn expression in NI (*p* = 0.0012) and higher GH in I and I + M (*p* = 0.0002); GHR1 and MyoD1 did not differ. Histomorphometry showed a significant interaction for villus height (interaction *p* = 0.004), with I + M having the highest post‐hoc values (I + M vs NI, *p* < 0.0001). Villus width increased due to independent effects of inversion (*p* < 0.0001) and supplementation (*p* = 0.0003). Muscle height exhibited an interaction (*p* = 0.0098) with I + M highest, while muscle width was greater in inverted and supplemented animals (*p* < 0.00001). Carbonylated protein levels showed a significant interaction (*p* = 0.0063); NI had the highest levels and microencapsulation reduced protein carbonylation in non‐inverted fish. Principal component analysis explained 71.8% of total variance and separate treatments along PC1, mainly associated with villus morphology, GH expression, and total length. Together, the results indicate that hormonal and nutritional modulation interact to enhance growth, tissue development, and redox homeostasis during early ontogeny of Nile tilapia.

## 1 Introduction

The larval stage of fish represents a period of high physiological plasticity, during which early stimuli can durably reprogram endocrine, metabolic, and structural pathways associated with growth, feed efficiency, and cellular homeostasis. This phenomenon, known as metabolic programming, involves the modulation of central hormonal axes, such as the GH–IGF axis, the regulation of myogenesis, and the functional organization of the gastrointestinal tract, whose initial alterations can be reflected in the productive phenotype throughout ontogeny (Yúfera et al. 2011; Moyano et al. 2018; Bertucci et al. 2019; Hou and Fuiman 2020; Navarro‐Guillén et al. 2024).

Hormonal sex inversion applied during the larval stage constitutes an intense endocrine stimulus that extends beyond simple sex determination (Opiyo et al. 2020; Silva et al. 2023). Early exposure to synthetic androgens affects systemic hormonal signaling, influencing the somatotropic axis, energy metabolism, and muscle tissue deposition (Leet et al. 2011; Baroiller et al. 2014). Although this practice is associated with higher growth rates in monosex male populations (Chávez‐García et al. 2020), it is an intervention applied during a critical period of physiological organization, with the potential to alter redox balance, metabolic dynamics, and nutrient utilization efficiency (Casetta et al. 2022; Holhorea et al. 2023).

In parallel, nutritional strategies based on organic acids and essential oils have been widely investigated as modulators of intestinal physiology and metabolism in fish. Organic acids act primarily by reducing luminal pH, modulating the microbiota, and producing short‐chain fatty acids, which exert trophic effects on the intestinal epithelium, favoring cell differentiation and absorptive efficiency (Ebrahimi et al. 2017; Fabay et al. 2022; Hoseini et al. 2022, 2023; Huang et al. 2022; da Silva et al. 2023; Hussein et al. 2023; Rasidi et al. 2024; Araujo Ferreira de Melo et al. 2025). Essential oils, in turn, possess antioxidant and antimicrobial properties, in addition to interfering with cellular signaling pathways associated with intestinal mucosal integrity and oxidative stress control. Consistent evidence indicates that these compounds can modulate growth, intestinal morphology, immune response, and redox stability in farmed fish (Alves de Azerêdo et al. 2012; Valladão et al. 2017; Souza et al. 2019; Valdivieso‐Ugarte et al. 2019; Abdel‐Latif et al. 2020; SOUZA et al. 2020; Alves Jesus et al. 2021; Huyben et al. 2021; Shourbela et al. 2021; Bandeira Junior et al. 2022; Özil et al. 2022; Zheng et al. 2024; Khalafalaa et al. 2025).

The microencapsulation of these bioactives represents a relevant advance, allowing greater stability of the compounds, protection against early degradation, and gradual release along the gastrointestinal tract (Mehta et al. 2022; Huang et al. 2023; Hamid et al. 2025). This characteristic is particularly important during the larval phase, when the digestive tract is developing and absorption efficiency is highly dependent on the structural and functional integrity of the intestinal epithelium (López‐Olmeda et al. 2021). Therefore, it is reasonable to propose that microencapsulated supplementation may exert more consistent effects on intestinal organization, energy availability, and resource allocation for somatic growth.

Despite the robust literature on the isolated effects of hormonal sex inversion and supplementation with organic acids and essential oils, the interaction between endocrine and nutritional stimuli during early metabolic programming remains poorly understood. Given this, the present study tested the hypothesis that the association between hormonal sex inversion and microencapsulated supplementation with organic acids and essential oils promotes synergistic effects on the zootechnical performance, expression of growth‐related genes, intestinal and muscle morphology, and oxidative status of Nile tilapia (*Oreochromis niloticus*) larvae, contributing to a more integrated understanding of endocrine‐nutritional programming mechanisms in fish.

## 2 Materials and Methods

### 2.1 Ethics

This study was approved by the Animal Ethics Committee (CEUA) of the State University of Maringá (UEM) under protocol 7650040820.

### 2.2 Experimental Design

The experiment was carried out according to a completely randomized design (CRD), arranged in a factorial scheme (2×2), involving both sex inversion and microencapsulated supplementation.

The experiment was carried out at the Aquaculture Laboratory of the State University of Maringá (UEM ‐ PR), using Nile tilapia larvae obtained from the university's Tilamax® selective breeding program, 6 days post‐hatching, with an initial body weight of approximately 0.012 ± 0.003 g. The animals were divided into four experimental groups: NI (non‐inverted animals), I (inverted animals), NI + M (non‐inverted animals supplemented with microencapsules) and I + M (inverted animals supplemented with microencapsules). Each experimental group was distributed across six tanks, totaling 24 experimental tanks (density of 100 larvae per tank). Each tank had a useful volume of 100 L and was connected to an independent semi‐recirculating aquaculture system equipped with an individual pump, mechanical filtration using porous media for suspended solid removal, and biological filtration with biofilter media supporting nitrifying bacteria. Water recirculation occurred individually within each tank, ensuring independence among experimental units. Continuous aeration was provided through air stones connected to a central blower. Daily water loss due to evaporation was approximately 25% and was compensated by the addition of fresh water to maintain water volume and water quality. The fish were adapted to the experimental conditions during a period of 1 day before the beginning of the experiment. Following this acclimation period, the animals were fed a specific diet eight times daily for 28 days.

### 2.3 Diets and Feeding

The basal diet was the commercial powdered diet ‘FISH 42% VITA C INTEGRADA®’. The microencapsulated organic acids and essential oils (supplied as ‘ENTERIFIN® M300 FISH’) and 17α‐methyltestosterone were added to this diet. The microcapsules (100 mg per kg⁻¹ of feed) and the hormone (60 mg per kg⁻¹ of feed, previously diluted in 98% absolute ethanol) were homogenously mixed into the powdered basal feed using a standard dry‐mixing procedure. The ethanol was allowed to evaporate completely before the feed was offered to the fish. The experimental diets were prepared weekly and stored at −20°C. Four experimental groups were formed, as follows:

1. Control diet (provided to NI group);
2. Diet with microcapsules (provided to NI + M group);
3. Control diet + 17α‐methyltestosterone (provided to I group);
4. Diet with microcapsules + 17α‐methyltestosterone (provided to I + M group).

The minimum nutritional levels of protein (42%) and ether extract (70 g kg^−^^1^) of the feed were ensured and confirmed through laboratory analysis using the methodology described by Silva and Queiroz 2006 (Supplementary Table S1). The exact contents of the diet and the composition of microcapsules are not provided as these are commercial products protected by patents.

The ingredients of the “FISH 42% VITA C INTEGRADA®” feed are: Wheat bran, ground whole corn, defatted corn germ bran, soy bean, beef and bone meal, chicken offal meal, chloride sodium, DL‐methionine l‐lysine, vitamins A, D3, E, K3, B1, B2, B3, B6, B7, B9, B12, C, pantothenic acid, choline chloride, sodium selenite, manganese monoxide, zinc oxide, iron sulfate, cobalt and copper sulfate, calcium, butylated hydroxide anisole (BHA), butylated hydroxide toluene (BHT), propionic acid, ammonium hydroxide. The complete formulation is proprietary information as it is a commercial product.

The microencapsulated product “ENTERIFIN®” has the following nutritional composition: monosodium citrate, fumaric acid, formic acid, sorbic acid, carvacrol, clove essential oil, thymol, vanillin, BHT (butylated hydroxide toluene), polyvinylpyrrolidone, hydroxypropyl and vegetable oil; with the following guaranteed levels: Citric acid (min) 40.0 g kg^−^^1^; formic acid (min) 80.0 g kg^−^^1^; fumaric acid (min) 30.0 g kg^−^^1^ and sorbic acid (min) 50.0 g kg^−^^1^). The complete formulation is proprietary information as it is a commercial product.

### 2.4 Water Quality

Water quality parameters were measured daily before the first feeding at 8 a.m. using portable instruments. Temperature (°C) and dissolved oxygen (mg L⁻¹) were measured with a YSI ProDSS oximeter, pH with a Hanna Instruments HI98107 pHep® meter, and electrical conductivity (µS cm⁻¹) and total dissolved solids (ppm) with a Hanna Instruments HI98319 conductivity meter. Ammonia (ppm) and nitrite (ppm) were monitored weekly using a colorimetric test kit (API Freshwater Master Test Kit) (Supplementary Table S2 and S3).

### 2.5 Performance

To determine weight gain (WG, Final Weight – Initial Weight), a total body weight was obtained for each experimental unit (tank) on days 0, 14, and 28. On day 0, this was achieved by weighing all larvae collectively and then dividing by the total count to obtain a mean initial weight. On days 14 and 28, the weight gain was assessed based on a biometry sample of 50 larvae per treatment (randomly selected from across the replicate tanks), which were individually weighed to calculate a mean. Standard length and total length were measured from digital photographs taken with a 1920px × 1080px resolution camera, for the same animal of WG. To determine the growth curve throughout the experimental period, ten animals were randomly selected and photographed weekly.

The larvae were subsequently measured using Image‐Pro Plus software. Feed efficiency (FE) was calculated as weight gain per unit of feed consumed was calculated for each tank as the relationship between the weight gain of the fish and the amount of feed provided. Feed intake was not directly measured but was precisely controlled and calculated based on the weekly biomass in each tank. Following the weekly biometrics, the total biomass per tank was determined. The daily feed ration was calculated and adjusted weekly according to the manufacturer's recommendations, considering the mean larval weight per tank and expressed as 12% of the total biomass (BW day^−^^1^).

### 2.6 Larval Euthanasia

Larvae were euthanized at sampling by rapid hypothermia through immersion in an ice‐water slurry (0°C–4°C) until opercular movement ceased, immediately followed by physical disruption of the brain (cranial concussion or spinal cord severance) as a secondary step to ensure death.

### 2.7 Histology of Muscle, Liver and Intestine

Samples of 15 larvae per treatment (5 larvae from each of 3 randomly selected tanks per treatment) were collected at the end of the feeding period. The larvae were fixed in Bouin's solution for 24 h, dehydrated in increasing concentrations of ethanol, cleared in xylene and embedded in paraffin blocks. 3 µm‐thick semi‐serial cross sections were obtained using a microtome and stained with hematoxylin and eosin (H&E) for morphometric assessment of muscle, liver and intestine. The slides were analyzed by light microscopy (Motic BA310E) using a digital microscope camera (Moticam 5.0 MP). The analysis and quantification of the microphotographed samples were performed in the Motic Images Plus 3.0 software.

The morphometric variables were measured and quantified based on clearly defined anatomical landmarks. For muscle morphometry, muscle height was defined as the vertical distance from the dorsal edge of the vertebral foramen to the apex of the dorsal musculature, while muscle width was measured as the perpendicular distance to this height axis, representing the lateral width of the epaxial musculature. For intestinal morphometry, villus height was measured from the apex of the villus to the villus‐crypt junction, and villus width was measured at the midpoint of the villus's height. For liver morphometry, hepatocyte density was assessed by counting the number of hepatocyte nuclei within a standardized area of 20,000 µm^2^. The specific variables quantified were muscle height and width, intestinal villus height and width, and hepatocyte count per 20,000 µm^2^.

### 2.8 Gene Expression

For gene expression analysis, six larvae from each treatment were euthanized and collected to analyze the expression of growth hormone (GH), growth hormone receptor (GHR1), Myostatin (Mstn) and myogenic differentiation (MyoD1)genes. Due to the small size of the larvae, the entire body (whole larval bodies) was used for RNA extraction to obtain sufficient quantity and quality of genetic material, providing an overview of the systemic gene expression. The samples were immediately placed in Eppendorf tubes, frozen in liquid nitrogen and stored in a freezer at −80°C until total RNA extraction.

A total of 75 mg of tissue was homogenized and extracted with TRIzol® (Invitrogen, Carlsbad, CA, USA) according to the manufacturer's instructions. All materials used were pretreated with RNase AWAY® (Invitrogen, Carlsbad, CA, USA), an RNase inhibitor. The extracted RNA (1 μL) was quantified in a Nanodrop 2000c spectrophotometer (Thermo Fisher ScientificTM‐). The purity and quality of RNA were confirmed by analyzing the absorbance ratios at 260 to 280 nm and 260 to 230 nm. RNA samples were treated with DNase I (Invitrogen, Carlsbad, CA, USA) to remove residual genomic DNA according to the manufacturer's instructions. Following total RNA extraction, complementary DNA (cDNA) was synthesized from 1 µg of total RNA using the SuperScriptTM III First‐Strand Synthesis SuperMix kit (Invitrogen Corporation, Brazil) according to the manufacturer's instructions. After this step, the samples were stored at −20°C until subsequent analysis.

Quantitative real‐time PCR (qPCR) was then performed using the PowerUP SYBR Green Master Mix (Applied Biosystems, Foster, CA, USA) on a StepOne Real‐Time PCR System version 2.3 (Applied Biosystems, Foster, CA, USA). The sequences of the GH (Tian et al. 2018), GHR1 1 (Claudino da Silva et al. 2019), Mstn and MyoD1 1 primers specific for the *Oreochromis niloticus* species are listed in Table 1. Three candidate endogenous control genes were evaluated: ubiquitin‐conjugating enzyme E (UBCE; GenBank accession DQ822452), elongation factor 1 alpha (EF1A; GenBank accession AB075952), and β‐actin (B‐ACT; GenBank accession AY116536). Amplification efficiency was determined for each primer pair, and expression stability was assessed across all experimental groups. β‐actin showed amplification efficiency within the acceptable range (90%–110%) and did not vary statistically among treatments; therefore, it was selected as the reference gene. All reactions were performed for the final volume of 10 μL in duplicates and the results are expressed in arbitrary units (AU). Dissociation curves were analyzed for any presence of primer dimers or non‐specific products.

**Table 1:** Sequence of *primers* used for real‐time PCR reactions.

Primer	Sequence (5′‐3′)	Accession No.
*GH*‐F	ACAGCCAGCGTTTGTTCTCCAT	MH936528.1
*GH*‐R	GGAAACTCCCAGGACTCAACCA	
*GHR1*‐F	TCTTGTATTTGGGACTGTGGG	AY973232.1
*GHR1*‐R	CGATGCCTTTGATTTTGGGTG	
*Mstn*‐F	CATCGAGATCAACGCTTTCG	AF197193
*Mstn*‐R	CTGGGGCCCTCTGAAATCTT	
*MyoD1*‐F	CTTCTACCCGGTGCTGGAG	AY337039
*MyoD1*‐R	CCAGACTGGAGACCACTGAAC	
*β ‐ACT*‐F	TGGTGGGTATGGGTCAGAAAG	AY116536
*β ‐ACT*‐R	TGTTGGCTTTGGGGTTCA	

### 2.9 Carbonyl Protein

Due to the small size of the larvae, the entire body (whole larval bodies) was used for this analysis. A total of 200 mg of Nile tilapia larvae from each groups was ground and added to 1000 µL of 0.05 mol L^−^^1^ phosphate buffer with 0.001 mol L^−^^1^ pH 6.7 ethylenediaminetetraacetic acid (EDTA). The solution was homogenized using a Van Potter homogenizer until complete dissociation. After that, this homogenate was centrifuged at 10,000 × g for 10 min at 4°C. The supernatant was collected in a clean Eppendorf tube and used as a sample.

For analysis of protein oxidation, we measured the formation of carbonyl derivatives using the 2,4‐dinitrophenyl‐hydrazine reagent [DNPH (Sigma‐Aldrich)], as described by Levine et al, 1994. The readings were taken at 370 nm using the Evolution^TM^ 300 UV‐VIS spectrophotometer (Thermo Fisher ScientificTM). The protein carbonyl concentration was determined using the Beer – Lambert equation, A = C × b × ε, in which A is the absorbance of the sample minus control absorbance, C is the protein carbonyl concentration, b is the light path length, and ε is the molar extinction coefficient (22.000 mol L^−^^1^ cm). The results were expressed as nmol of carbonyl protein per mg of protein. The Bradford method (1976) was used to determine the total protein content and correct the protein carbonyl results.

### 2.10 Statistical Analysis

All statistical analyses were performed using R software (version 4.3.2; R Core Team, 2023). Model residuals were assessed for normality (Shapiro–Wilk test) and homoscedasticity (Levene's test). When both assumptions were met, data were analyzed using a two‐way analysis of variance (ANOVA) with sex inversion and microencapsulated supplementation as fixed factors, including their interaction, followed by Tukey's HSD post‐hoc test for significant effects. If residuals deviated from normality but homoscedasticity was maintained, data were either transformed (logarithmic or square root) and reanalyzed or subjected to a nonparametric aligned rank transform (ART) procedure for factorial comparisons with contrast analyses. When normality was satisfied but variances were heterogeneous, a two‐way ANOVA with robust covariance estimation (White's adjustment) was employed. When both assumptions were violated, a nonparametric Scheirer–Ray–Hare (SRH) test was applied, followed by Dunn's post‐hoc test with Bonferroni correction for significant main effects.

Weight gain, total length, muscle morphometry (height and width), and intestinal villi morphometry (height and width) were analyzed using the approaches described above. For repeated measurements over time (days 0, 7, 14, 21, 28), a repeated measures ANOVA was employed. Whenever significant main or interaction effects were detected (*p* < 0.05), data were analyzed separately for each time point using one‐way ANOVA followed by Tukey's HSD post‐hoc test for multiple comparisons among treatment groups. For cases where only two groups were compared (e.g., inversion vs. no inversion), Welch's *t*‐test or the Mann–Whitney test was applied according to data distribution.

Expression levels of growth hormone (GH), growth hormone receptor (GHR1), myostatin (MSTN), and myogenic differentiation factor (MyoD1)were analyzed using the 2^−^^ΔΔCt^ method. Statistical differences were determined using two‐way ANOVA with sex inversion and supplementation as fixed factors. For genes showing significant effects (GH and MSTN), Tukey's HSD test was applied for post‐hoc comparisons among treatment groups. Hepatocyte counts, intestinal villi dimensions, muscle morphometry, and protein carbonylation levels were analyzed using two‐way ANOVA, followed by Tukey's HSD post‐hoc test when significant interactions between main factors were detected. Total length growth data were fitted to three non‐linear models (Gompertz, Logistic, and von Bertalanffy) using the Levenberg‐Marquardt algorithm via the nlsLM function in the minpack. lm package (version 1.2‐3). Model parameters were estimated automatically: A (asymptotic maximum length), k (growth rate), tᵢ (time at inflection point), and t₀ (theoretical time at length zero). Model selection was based on the lowest Akaike Information Criterion (AIC).

Multivariate analysis was performed on eight standardized morphometric and gene expression variables: weight, total length, muscle height, muscle width, villus height, villus width, MSTN expression, and GH expression. Data were scaled to unit variance prior to analysis using the prcomp function. Results were visualized using the factoextra package (version 1.0.7), with treatment group separation indicated by 95% confidence ellipses. Variable contributions to principal components were calculated as the squared cosine of variable coordinates. All results are reported as mean ± standard deviation (SD). Statistically significant differences (*p* < 0.05) between treatment groups are indicated in figures by distinct lowercase letters, based on post‐hoc comparisons. All figures were created using the ggplot2 package (version 3.4.4), and selected illustrations were refined with BioRender. com for clarity. A detailed decision flow, specifying the assumption tests and statistical procedures applied for each response variable, is provided in Supplementary Table S4.

## 3 Results

The results of the statistical analysis for each measured variable are presented below. The specific statistical approach applied to each variable was determined by diagnostic checks of model assumptions (normality and homoscedasticity). A complete decision workflow detailing the assumption test results and the specific method applied to each variable is provided in Supplementary Table S4 (Statistical Workflow).

**Figure 1:**
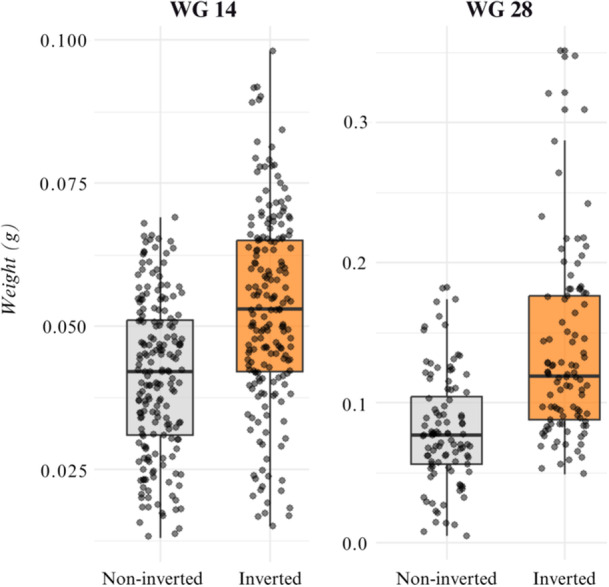
Boxplot of tilapia larval weight (g) at different sampling times (D14—Welch's *p* < 0.001 and D28—Mann–Whitney test; *p* < 0.001) for treatments NI (without sex inversion) and I (with sex inversion). [Color figure can be viewed at wileyonlinelibrary.com]

For weight gain at 14 days (WG14), a significant effect of inversion was observed (*p* < 0.001), while microencapsulated supplementation (*p* = 0.2459) and the interaction (*p* = 0.1100) were not significant. For WG28, a significant effect of sex inversion was found (H = 46.914; *p* < 0.001), with no effect of supplementation (H = 0.010; *p* = 0.920) or interaction (H = 0.142; *p* = 0.707) (Figure 1).

**Figure 2:**
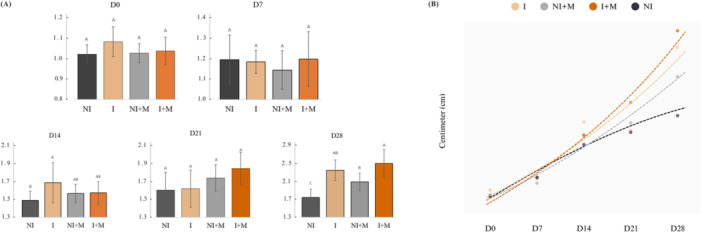
(a) Mean ± standard deviation of total length (cm) of larval tilapias at different sampling times (D0, D7, D14, D21, and D28) for the different treatments: NI (without sex inversion), I (with sex inversion), NI + M (without sex inversion + microencapsulated nutritional supplement), and I + M (with sex inversion + microencapsulated nutritional supplement). Different letters indicate statistical differences among treatments within each time point, according to Tukey's test (*p* < 0.05). (b) Growth curve fitting over 28 days for the different treatments. [Color figure can be viewed at wileyonlinelibrary.com]

For total length, no significant differences between treatments were detected on days D0 (*p* = 0.099), D7 (*p* = 0.479), and D21 (*p* = 0.060). A significant treatment effect was observed on D14 (*p* = 0.039) and D28 (*p* < 0.001) (Figure 2a).

The growth data fitting revealed distinct patterns among the treatments. Groups I, I + M, and NI + M were better described by the logistic model, while the NI treatment was more adequately fitted by the Gompertz model. Among the logistic treatments, NI + M showed the highest asymptote (A = 198.32), indicating a greater potential for final performance, although with a lower growth rate (k = 0.0053) and a later inflection time (ti = 314.77 days). The I + M group exhibited a high asymptote (A = 123.79) and a moderate growth rate (k = 0.0332), with an intermediate inflection time (ti = 159.78 days). The I treatment showed intermediate values (A = 76.57; k = 0.0284; ti = 145.47 days), representing moderate growth in both rate and magnitude. On the other hand, The NI treatment, best fitted by the Gompertz model, displayed the lowest asymptote (A = 2.00) and an early inflection time (ti = 0.11 days), despite a high growth rate (k = 0.072).

**Figure 3:**
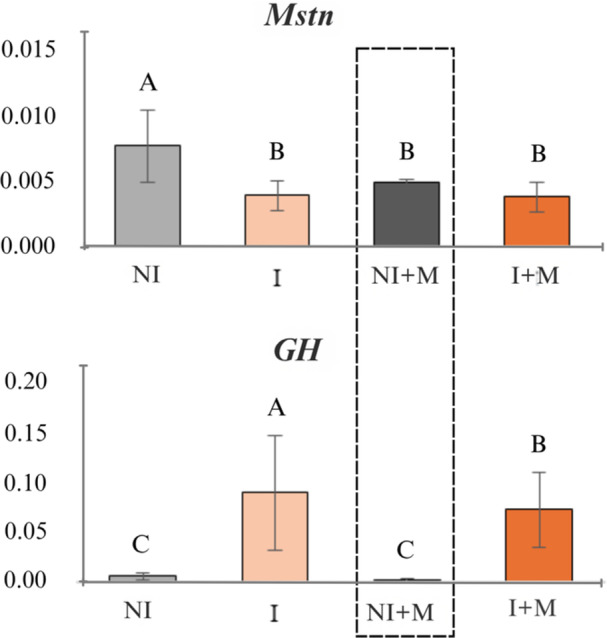
Gene expression of Mstn (myostatin) and GH (growth hormone) in larval tilapia under different experimental conditions: NI (no sex inversion), I (sex inversion), NI + M (no sex inversion + microencapsulated nutritional supplement), and I + M (sex inversion + microencapsulated nutritional supplement). Uppercase letters in parentheses indicate significant differences (Tukey HSD for Mstn, robust multiple comparisons with White adjustment for GH, *p* < 0.05). [Color figure can be viewed at wileyonlinelibrary.com]

Gene expression analysis revealed a significant difference among treatments for *Mstn* (*p* = 0.0012), with the highest expression in NI. No differences were found for *GHR1* (*p* = 0.7838). A significant treatment effect was observed for *GH* expression (*p* = 0.0002), where treatment I had the highest expression, followed by I + M, while NI and NI + M showed the lowest expressions. No significant differences were observed for *MyoD1* (*p* = 0.1025) (Figure 3).

**Figure 4:**
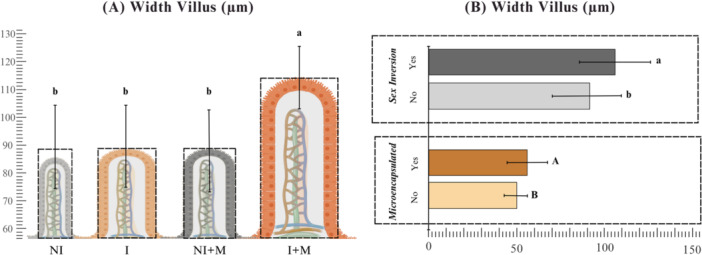
Height and width of intestinal villi of Nile tilapia larvae subjected to sex inversion (I) and/or microencapsulated supplementation (M). (A) Villus height (µm). Different letters above the bars indicate statistically significant differences after interaction unfolding by Tukey's test (*p* < 0.05). (B) Villus width (µm). Uppercase letters in front of the bars indicate significant differences for the microencapsulated supplementation factor (Dunn's test, *p* < 0.001), while lowercase letters indicate significant differences for the sex inversion factor (Dunn's test, *p* < 0.0001). Values are expressed as mean ± standard deviation. [Color figure can be viewed at wileyonlinelibrary.com]

Hepatic histomorphometry showed no differences in hepatocyte number (*p* = 0.2686). For villus height, significant main effects of sex inversion (*p* = 0.00146) and supplementation (*p* = 0.00053), and a significant interaction (*p* = 0.0040) were found. The I + M group showed the greatest height, differing from all others (*p* < 0.0001) (Figure 4A). For villus width, both sex inversion and supplementation had significant effects (*p* < 0.001 for both), with no interaction (*p* = 0.2390). Inverted animals had greater width than non‐inverted (*p* < 0.0001), and supplemented groups exceeded non‐supplemented ones (*p* = 0.0003) (Figure 4B).

**Figure 5:**
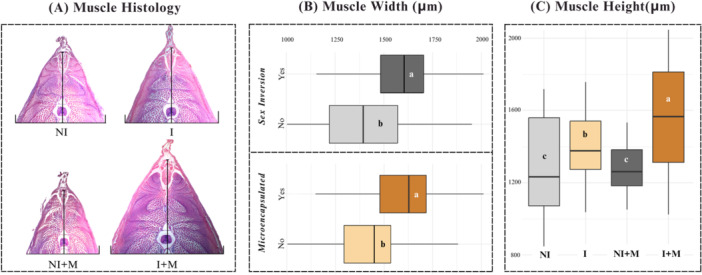
(A) Transverse histological sections of the dorsal white muscle region of larval tilapia subjected to four different treatments: NI (no sex inversion), I (with sex inversion), NI + M (no sex inversion + microencapsulated supplement) and I + M (with sex inversion + microencapsulated supplement). Muscle width (B) and height (C) among experimental groups. Different letters indicate statistical differences between treatments according to Dunn's test with Bonferroni correction (*p* < 0.05). Data expressed in boxplots showing medians, quartiles and ranges. [Color figure can be viewed at wileyonlinelibrary.com]

A significant interaction was found for muscle height (*p* = 0.0098). The I + M group showed the greatest height, followed by I, while NI and NI + M showed the lowest (*p* < 0.0001). For muscle width, significant effects of inversion (*p* < 0.0001) and microencapsulation (*p* < 0.0001) were observed, with no interaction (*p* = 0.3435). Inverted and supplemented animals presented greater muscle width (*p* < 0.0001) (Figure 5).

**Figure 6:**
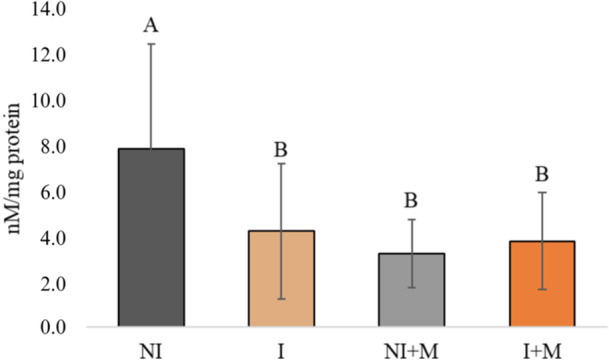
Carbonylated protein of larval tilapia under different experimental conditions: NI (no sex inversion), I (with sex inversion), NI + M (no sex inversion + microencapsulated supplement) and I + M (with sex inversion + microencapsulated supplement). Different letters indicate statistically significant differences between treatments according to simple effects analysis of two‐way ANOVA with White's adjustment (*p* < 0.05). Data expressed as means ± standard deviation. [Color figure can be viewed at wileyonlinelibrary.com]

For carbonylated proteins, a significant interaction between sex inversion and microencapsulation was found (*p* = 0.0063). Microencapsulation significantly reduced protein carbonylation in non‐inverted fish (*p* < 0.0001) but had no effect in inverted fish (*p* = 0.8007). The NI group showed the highest levels (Figure 6).

**Figure 7:**
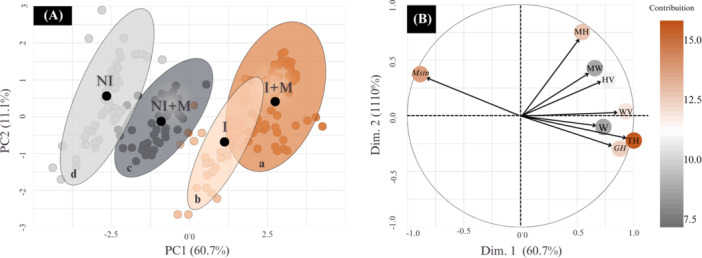
Principal Component Analysis (PCA) of morphometric parameters and gene expression in larval tilapia under different experimental conditions: NI (no sex inversion), I (with sex inversion), NI + M (no sex inversion + microencapsulated nutritional supplement) and I + M (with sex inversion + microencapsulated nutritional supplement). (A) 95% confidence ellipses of experimental group separation; distinct letters within ellipses (a–d) indicate significant differences (*p* < 0.05) between group centroids on the PC1 axis, according to Bonferroni post‐hoc test. (B) PCA biplot: Variable contributions to the first two principal components. Vectors indicate the direction and intensity of each variable's contribution. Vector length is directly proportional to its importance within the component. GH = growth hormone expression, HV = villus height, MH = muscle height, MW = muscle width, Mstn = myostatin expression, TH = total height, W = weight, WV = villus width. [Color figure can be viewed at wileyonlinelibrary.com]

PCA revealed a clear separation between experimental groups in the multivariate space (Figure 7). The first two principal components (PC1 and PC2) together explained 71.76% of the total data variance. The main source of variation (PC1 −60.68%) was primarily driven by total height (contribution = 17.63%), villus width (16.02%), and GH expression (15.02%). PC2 (11.08%) was predominantly influenced by muscle height (63.99%). The analysis demonstrated distinct clustering patterns among treatments in the multidimensional space.

## 4 Discussion

The present study evaluated the effects of sex inversion and microencapsulated supplementation on the performance, gene expression, and morphology of Nile tilapia larvae. Weight gain and length were consistently higher in the sex‐reversed groups (I and I + M), especially at the end of the experimental period (D28). This superior growth can be explained by the establishment of a male (androgenic) endocrine profile, which promotes an anabolic metabolic state. Physiologically, androgens stimulate muscle protein synthesis, positively modulate the growth hormone/insulin‐like growth factor‐1 (GH/IGF‐1) axis, and direct dietary energy preferentially toward somatic growth, in contrast to females, which in later stages divert resources toward gonadal development (Larsen et al. 2004; Dubois et al. 2012; SCULTHORPE et al. 2012). These findings corroborate previous studies indicating higher feed conversion efficiency and growth in males or sexually manipulated individuals (Chávez‐García et al. 2020; Silva et al. 2023). From a nutritional standpoint, these results indicate that sex inversion establishes a physiological background that can potentiate the effects of functional dietary strategies, such as microencapsulated supplementation. Therefore, from a production standpoint, these results reinforce that sex inversion should be viewed not only as a reproductive management tool but as a key physiological primer that maximizes the return on investment in advanced nutritional strategies like microencapsulation.

The superior fit of the Logistic model to the I, I + M, and NI + M groups reveals that both sex inversion and microencapsulated supplementation promote sustained growth patterns, with more prolonged growth phases—a profile associated with greater feed efficiency and lower metabolic stress in aquaculture (Zullinger et al. 1984; Ansah and Frimpong 2015). Physiologically, these treatments modulate distinct energy allocation strategies. The NI + M group, with its exceptional final potential (A = 198.32) achieved slowly, illustrates how supplementation alone can redirect metabolism toward extreme nutritional efficiency, prioritizing gain in quality (potential) over speed (Ricker 1975). In contrast, the I + M group combines a good final potential with a robust growth rate, demonstrating the synergy between the male anabolic profile and functional nutrition. In clear contrast, the fit of the Gompertz model to the NI group exposes an inefficient “boom and bust” strategy: an explosive initial growth (k = 0.072) that prematurely exhausts developmental potential (A = 2.00), a classic *trade‐off* where speed compromises efficiency and final outcome (Tjørve and Tjørve 2017). This analysis has direct practical implications for larviculture. The balanced profile of the I + M group confirms it as the most recommended integrated protocol, maximizing zootechnical return. However, the result of the NI + M group is equally valuable, as it demonstrates that microencapsulated supplementation is a tool capable of rescuing growth potential even in non‐inverted populations, converting a trajectory of early exhaustion into one of high efficiency and sustainability throughout the production cycle.

The evaluation of gene expression provided a partial yet clarifying molecular rationale for the distinct growth profiles. Groups exhibiting sustained growth (I, I + M) showed higher expression of GH, directly stimulating the hepatic GH‐IGF‐1 axis to promote muscle hyperplasia and hypertrophy (Dubois et al. 2012; SCULTHORPE et al. 2012). Conversely, the limited‐growth profile of the NI group was associated with elevated expression of Mstn, a known inhibitor of the anabolic Akt/mTOR pathway that suppresses protein synthesis (Liu et al. 2020). The pivotal finding emerged from the NI + M group, where microencapsulated supplementation suppressed Mstn expression to levels equivalent to those in sex‐reversed groups. This indicates that the supplement acts not merely as a nutrient source but as a transcriptional modulator, actively removing a molecular constraint on growth. Collectively, these results reinforce the established paradigm of GH and Mstn as central, antagonistic regulators of somatic growth in vertebrates (McPherron and Lee 1997; Matsakas and Diel 2005; Ndandala et al. 2022). In practice, our results suggest a targeted protocol: assess Mstn expression in larvae to identify batches prone to limited growth and then apply microencapsulated supplementation as a specific corrective intervention. This optimizes the supplement's cost‐benefit ratio, transforming it into a solution for a well‐defined physiological bottleneck.

The histomorphometric analysis identified the structural foundations of the growth phenotypes. Integrated alterations were observed in intestinal villus height and muscle fiber height, with the I + M group exhibiting the highest values. Additive effects were recorded for the width of both tissues, while the hepatic structure remained unchanged. These results constitute the final phenotypic manifestation of the previously described molecular profiles. The increase in villi represents an adaptation of absorptive capacity, ensuring a greater nutrient flux (Scocco and De Felice 2025). The muscular expansion, particularly in the I + M group, translates this nutritional efficiency into somatic deposition (Silva et al. 2009; Zhang et al. 2022), while the absence of hepatic hyperplasia suggests growth sustained by functional optimization. Collectively, these findings support a model in which the endocrine‐nutritional alignment channels gene modulation (GH ↑ , Mstn ↓ ) into a coordinated remodeling of the absorption and deposition tissues. This model aligns with the consensus that the expansion of villus surface area correlates with greater digestive efficiency (Walton et al. 2016), and muscle hypertrophy is a direct marker of protein anabolism (Dubois et al. 2012). Therefore, the I + M protocol bases its superior performance on an integrated morphophysiological reconfiguration, simultaneously optimizing nutrient uptake and utilization. This positions it as a precision zootechnical intervention capable of converting molecular permissiveness into a sustainable productive advantage.

The analysis of carbonylated proteins revealed higher oxidative damage in the NI group, indicating greater physiological stress. Nutritionally, this oxidative burden represents a metabolic cost that diverts energy from growth and compromises cellular homeostasis through macromolecular damage (Grant 2008; Krishnamurthy et al. 2024; Chandimali et al. 2025). The lower oxidative damage in supplemented and/or sex‐reversed groups suggests an enhanced physiological resilience. Notably, this finding contrasts with reports that sex inversion itself can activate the stress axis (Todd et al. 2019; Goikoetxea et al. 2022). Our results imply that under controlled hatchery conditions, the endocrine homogenization from sex inversion, coupled with nutritional modulation, may attenuate metabolic variability and promote a more stable redox state. Therefore, beyond explaining performance differences, oxidized protein levels serve as a functional biomarker of hatchery stress. The I + M protocol thus emerges not only as a growth‐promoting strategy but as a means to reduce latent metabolic overhead, channeling a greater share of dietary energy into biomass production.

The integrated view from PCA corroborates and unifies the variations observed among treatments. The clear separation of groups, primarily along PC1 (60.68% of the variance), defines distinct global phenotypes. Significantly, the variables contributing most to this separation were total length, intestinal villus height, and GH expression. This triad (growth, absorption, regulation) forms the central axis of the treatment response, as documented in fish farming (Stevens and Devlin 2000; Liu et al. 2014; Huyben et al. 2021). Although PCA does not establish causality, the observed association is physiologically coherent with the proposed model, wherein higher GH expression stimulates both somatic growth and intestinal development, optimizing nutrient absorption (Petro‐Sakuma et al. 2020). The position of the I + M group at the positive extreme of PC1, aligned with the vectors of this triad, consolidates the study's central interpretation. Therefore, the multivariate analysis not only validates individual differences but identifies morphophysiological integration as the signature of the superior phenotype. The I + M protocol, by synergistically leveraging sex inversion and supplementation, orchestrates this integration, maximizing the conversion of nutrients into biomass and setting a new benchmark for zootechnical efficiency in tilapia larviculture.

## 5 Conclusion

The combination of sex inversion and microencapsulated nutritional supplementation promoted synergistic effects on the growth, intestinal and muscle morphology, and physiological status of Nile tilapia larvae. This combined treatment resulted in higher GH expression, more developed intestinal villi, hypertrophied muscle fibers, and lower accumulation of carbonylated proteins, establishing a phenotype of high metabolic efficiency and low oxidative stress. Supplementation alone also positively modulated gene expression and muscle structure, demonstrating potential applicability even in non‐inverted production systems. In an integrated manner, the results suggest that controlled‐release nutritional strategies can potentiate the zootechnical effects of sexual manipulation, optimizing the initial performance and welfare of the fish. Future studies should assess the persistence of these effects in later growth stages and their influence on reproductive and productive indicators.

## Funding

The authors received no specific funding for this work.

## Ethics Statement

The animal study protocol was approved by the Ethics Committee on Animal Use of the State University of Maringá (CEUA/UEM protocol 7650040820). All procedures were conducted in accordance with the Brazilian National Council for the Control of Animal Experimentation (CONCEA) guidelines and the ARRIVE guidelines.

## Conflicts of Interest

In accordance with the journal's policy, all authors have reviewed this policy collectively. We declare that we have no conflicts of interest to disclose, whether financial, proprietary, consulting, or of any other nature, that are directly relevant to the content of this manuscript.

## Supporting information

supplementary data.

## Data Availability

Data and Code Availability: In accordance with the FAIR principles (Findable, Accessible, Interoperable, and Reusable) that guide open science, we make the analytical resources of this study fully available. The complete set includes both the raw data organized in data frames and the detailed statistical script containing all analyses performed. This approach ensures not only methodological transparency but also full reproducibility and the potential for creative reuse by the scientific community. Resources available at: https://docs.google.com/document/d/1ZsIkMq0TFxxWVWS6lN9PQbQJ-_Twx8HY/edit?usp=sharing&ouid=111966773610880670816&rtpof=true&sd=true.
