# Supplementary material for: Endocrine‐Nutritional Synergy Between Sex Inversion and Microencapsulated Bioactives Enhances Growth, Intestinal Development, and Oxidative Stability in Nile Tilapia Larvae
Source: J Anim Physiol Anim Nutr (Berl). 2026 Feb 10;110(3):339–50. doi: 10.1111/jpn.70046 (PMC13172653; doi:10.1111/jpn.70046)
Supplement: Supplementary file 1 — supplementary data. [file JPN-110-339-s001.docx]

1. Analysis of minimum nutritional levels of protein (42%) and ether extract (70 g kg^-1^) of the feed

| **Feed** | **Crucible + Wet Sample Weight (g)** | **Crucible Weight (g)** | **Wet Sample Weight (g)** | **Crucible + Dry Sample Weight (g)** | **Moisture (%)** | **Average Moisture (%)** | **Sample Weight** | **H₂SO₄ Titration** | **H₂SO₄ Titration (Actual)** | **Total Nitrogen (%)** | **Protein (%)** | **Average Protein (%)** | **Sample Weight** | **Cartridge Weight (dry)** | **Cartridge Weight (fat-free)** | **Ether Extract (%)** | **Average Ether Extract (%)** |
| --- | --- | --- | --- | --- | --- | --- | --- | --- | --- | --- | --- | --- | --- | --- | --- | --- | --- |
|  |  |  |  |  |  |  |  | Blank = | 0.2 |  |  |  | X | X | X | X |  |
| 1 | 197.369 | 185.711 | 11.658 | 196.053 | 112.884 | **11.27** | 0.1067 | 50.2 | 50.00 | 6.56 | 41.00 | **43.93** | 16.085 | 24.565 | 23.251 | 8.17 | **8.56** |
| 2 | 175.171 | 162.798 | 12.373 | 173.738 | 115.817 |  | 0.1033 | 52.7 | 52.50 | 7.12 | 44.47 |  | 14.054 | 25.456 | 24.093 | 9.70 |  |
| 3 | 164.203 | 153.361 | 10.842 | 163.010 | 110.000 |  | 0.1036 | 52.1 | 51.90 | 7.01 | 43.83 |  | 14.963 | 24.853 | 23.773 | 7.22 |  |
| 4 | 180.020 | 169.968 | 10.052 | 178.963 | 105.153 |  | 0.1029 | 53.7 | 53.50 | 7.28 | 45.49 |  | 14.329 | 26.545 | 25.315 | 8.59 |  |
| 5 | 162.569 | 151.914 | 10.655 | 161.294 | 119.662 |  | 0.1014 | 52.2 | 52.00 | 7.18 | 44.87 |  | 15.036 | 24.345 | 22.971 | 9.14 |  |

1. Daily Water Parameters

| **Day** | 1 | 2 | 3 | 4 | 5 | 6 | 7 | 8 | 9 | 10 | 11 | 12 | 13 | 14 | 15 | 16 | 17 | 18 | 19 | 20 | 21 | 22 | 23 | 24 | 25 | 26 | 27 | 28 |
| --- | --- | --- | --- | --- | --- | --- | --- | --- | --- | --- | --- | --- | --- | --- | --- | --- | --- | --- | --- | --- | --- | --- | --- | --- | --- | --- | --- | --- |
| **Temperature (°C)** | 27.75 | 27.43 | 27.82 | 28.26 | 27.38 | 27.38 | 28.29 | 27.88 | 27.27 | 27.77 | 27.27 | 27.27 | 27.62 | 26.54 | 26.64 | 27.22 | 26.99 | 27.66 | 27.05 | 26.79 | 28.23 | 27.39 | 27.53 | 26.79 | 27.23 | 27.56 | 26.92 | 27.69 |
| **Dissolved Oxygen (mg/L)** | 5.62 | 5.71 | 5.62 | 6.36 | 5.8 | 5.48 | 6.05 | 5.43 | 5.86 | 5.21 | 5.4 | 5.86 | 6.02 | 5.85 | 5.77 | 5.71 | 5.36 | 5.58 | 5.66 | 6.12 | 5.9 | 5.27 | 5.9 | 5.68 | 5.6 | 5.98 | 6.11 | 6.08 |
| **pH** | 7.32 | 7.37 | 7.43 | 7.5 | 7.35 | 7.38 | 7.29 | 7.28 | 7.48 | 7.54 | 7.39 | 7.5 | 7.44 | 7.34 | 7.44 | 7.55 | 7.4 | 7.56 | 7.14 | 7.48 | 7.41 | 7.37 | 7.41 | 7.2 | 7.38 | 7.44 | 7.55 | 7.35 |
| **Conductivity (µS/cm)** | 434 | 440 | 468 | 457 | 439 | 460 | 452 | 469 | 436 | 443 | 442 | 421 | 456 | 455 | 450 | 445 | 422 | 442 | 443 | 434 | 447 | 458 | 488 | 453 | 455 | 449 | 412 | 449 |
| **TDS (ppm)** | 221 | 245 | 218 | 223 | 220 | 208 | 231 | 228 | 228 | 211 | 234 | 206 | 226 | 242 | 210 | 214 | 221 | 215 | 204 | 221 | 209 | 225 | 211 | 235 | 212 | 217 | 228 | 208 |

1. Weekly Water Parameters

| **Day** | **Ammonia (ppm)** | **Nitrite (ppm)** |
| --- | --- | --- |
| Day 0 | 0.213 | 0.068 |
| Day 7 | 0.161 | 0.091 |
| Day 14 | 0.156 | 0.091 |
| Day 21 | 0.098 | 0.067 |
| Day 28 | 0.129 | 0.075 |

1. Statistical Workflow

| **Variable / Dataset** | **Normality (p-value)** | **Homoscedasticity (p-value)** | **Statistical Method Applied** | **Post-hoc / Contrast Analysis** | **Key Findings (Summary)** |
| --- | --- | --- | --- | --- | --- |
| **Weight Gain (WG14)** | OK (0.0786) | Violated (0.0092) | Two-way ANOVA with White's adjustment | Not specified for main factors | Significant effect of inversion (p < 0.001). |
| **Weight Gain (WG28)** | Violated (< 0.001) | Violated (0.00036) | SHR | Mann-Whitney U test | Significant effect of inversion (p < 0.001). |
| **Total Length (D0)** | OK (0.610) | OK (0.338) | Two-way ANOVA | Tukey's test | No significant differences (p = 0.099). |
| **Total Length (D7)** | Violated (0.028) | OK (0.399) | Aligned Rank Transform | Not significant | No significant differences (p = 0.479). |
| **Total Length (D14)** | OK (0.179) | OK (0.117) | Two-way ANOVA | Tukey's test | Significant effect (p = 0.039). I > I+M ≈ NI+M > NI |
| **Total Length (D21)** | OK (0.326) | OK (0.559) | Two-way ANOVA | Tukey's test | No significant differences (p = 0.060). |
| **Total Length (D28)** | OK (0.151) | OK (0.627) | Two-way ANOVA | Tukey's test | Significant effect (p < 0.001). |
| **Mstn Expression** | OK (0.488) | OK (0.2476) | Two-way ANOVA | Tukey's HSD | Significant differences (p = 0.0012). NI had highest expression. |
| **GHR Expression** | OK (0.4216) | OK (0.3337) | Two-way ANOVA | Not significant | No significant differences (p = 0.7838). |
| **MyoD Expression** | OK (0.4114) | Violated (0.0106) | Two-way ANOVA | Not significant | No significant differences (p = 0.1025). |
| **GH Expression** | OK (0.4501) | Violated (0.0008) | Two-way ANOVA | Robust comparisons (White) | Significant differences (p = 0.0002). I > I+M > NI ≈ NI+M |
| **Hepatocyte Number** | Assumed met | Assumed met | Two-way ANOVA | Not performed | No significant differences (p = 0.2686). |
| **Villus Height** | OK (> 0.05) | OK (0.2535) | Two-way ANOVA | Tukey's test | Significant interaction (p = 0.0040). I+M > all others. |
| **Villus Width** | Violated (0.0012) | Violated (< 0.0001) | SHR | Dunn's test with Bonferroni | Significant main effects of inversion and supplementation. I > NI; M > No M |
| **Muscle Height** | Violated (0.0016) | Violated (< 0.0001) | SHR | Dunn's test with Bonferroni | Significant interaction (p = 0.0098). I+M > I > NI ≈ NI+M |
| **Muscle Width** | Violated (0.0001) | Violated (< 0.0001) | SHR | Dunn's test with Bonferroni | Significant main effects of inversion and supplementation. I > NI; M > No M |
| **Carbonylated Protein** | OK (0.2453) | Violated (0.0066) | Two-way ANOVA | Simple Effects Analysis | Significant interaction (p = 0.0063). Microencapsulation reduced carbonylation only in NI. |

SHR: Nonparametric Scheirer–Ray–Hare test
